# Transient renal tubular injury among children and adolescents during diabetic ketoacidosis: severity, renal perfusion, and urinary netrin- 1 interplay

**DOI:** 10.1007/s00431-025-06145-1

**Published:** 2025-05-07

**Authors:** Randa M. Matter, Dina E. Sallam, Sara I. Taha, Shrouk M. Awadallah, Rana Khamees, Nouran Y. Salah

**Affiliations:** 1 Pediatric Department, Faculty of Medicine, Ain Shams University, Cairo, Egypt; 2 Pediatric and Pediatric Nephrology Department, Faculty of Medicine, Ain Shams University, Cairo, Egypt; 3 Clinical Pathology Department, Faculty of Medicine, Ain Shams University, Cairo, Egypt; 4 Radiodiagnosis and Interventional Radiology Department, Faculty of Medicine, Ain Shams University, Cairo, Egypt; 5 Faculty of Medicine, Ain Shams University, Cairo, Egypt

**Keywords:** Children and adolescents, Moderate and severe DKA, Tubulopathy, AKI, Netrin

## Abstract

Acute kidney injury (AKI) has been reported during diabetic ketoacidosis (DKA). Evidence regarding tubulopathy is less established, with its pathophysiology, risk determinants, and short-term outcome to be unraveled. Hence, this study aimed to assess renal tubular functions during DKA, its short-term outcome, and its relation with urinary Netrin-1, DKA severity, and renal perfusion indices. Forty children and adolescents (20 with moderate and 20 with severe DKA) were assessed for urine output (UOP), blood pressure, blood glucose, HbA1c, and urinary Netrin-1, with calculation of serum osmolality and estimated glomerular filtration rate (eGFR). Renal pulsatility and resistivity indices were assessed by renal duplex. Reevaluation was done on days 3 and 14. Sixteen children and adolescents had tubular proteinuria during DKA (40%). Their mean urine output (UOP) during DKA was 14.03 cc/kg/h, and their mean urinary Netrin-1 was 836.9 ng/ml. A significant improvement was observed in the UOP, protein/creatinine ratio, urinary netrin, and serum osmolality after the resolution of DKA accompanied by a significant decrease in renal resistivity and pulsatility indices (*p *< 0.05). A significant positive correlation was found between tubular proteinuria during DKA and urinary Netrin-1, renal pulsatility, and resistivity indices (*p *< 0.05). Multivariate regression analysis revealed that serum PH and urinary Netrin-1 were the most significant independent variables associated with tubular proteinuria among children and adolescents during DKA.

*Conclusion*: Transient renal tubulopathy occurs during DKA manifested by tubular proteinuria, polyuria, and hypokalemia; that is correlated with DKA severity, renal perfusion indices, and urinary Netrin-1 and reversible by day 14 post-DKA. Netrin-1 could serve as a potential therapeutic target for DKA-associated tubulopathy.

**Table:** 

**What is Known:** *• Diabetic ketoacidosis (DKA) is a severe acute complication of diabetes mellitus, with negative effect on multiple body organs.* *• Studies increasingly suggest acute kidney injury during DKA, however, data about renal tubular injury during DKA, it's pathophysiology, risk determinants and short term outcomes are still unclear.*	
**What is New:** *• Transient renal tubulopathy was reported in 40 % the studied children and adolescents during DKA manifested by proteinuria, polyurea, and hypokalemia.* *• This tubulopathy that was correlated with DKA severity, renal perfusion indices and urinary Netrin-1 and totally reversible by day 14 post DKA.*

**Graphical Abstract:**

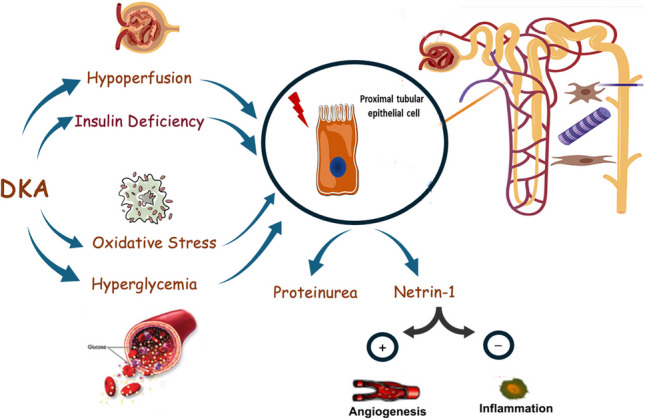

## Introduction

People with type 1 diabetes mellitus (T1DM) are at risk of developing both acute and chronic complications. Diabetic ketoacidosis (DKA) is one of the severe acute complication involving a series of closely related body fluids, electrolytes, and acid–base derangements [1]. DKA is associated with marked hyperglycemia, leading to glucose-induced osmotic diuresis, which, together with vomiting from ketosis, leads to volume depletion, predisposing to acute kidney injury (AKI) [2].

Diabetes-associated chronic kidney disease (CKD) has longly gained significant attention being a leading cause of diabetes morbidity and mortality [3]. Recently, DKA-associated AKI is increasingly recognized [4]. AKI, with or without renal tubular injury, has been reported in 43.8% of children newly diagnosed with T1DM, and 65% of children newly presenting with DKA, with further increase of this frequency up to 81% in cases of recurrent DKA episodes. Although both AKI and tubular injury are reversible, they have been recognized as major contributors to short-term poor DKA outcomes as well as long-term poor outcomes, since even a mild episode of AKI can double the future risk of CKD [5]. Therefore, early recognition and management of renal tubular injury and AKI are crucial for optimal diabetes management.

Currently, the diagnosis of renal impairment encompasses a reduction in glomerular filtration rate and rise in serum creatinine with or without oliguria, as described by the Kidney Disease/Improving Global Outcome (KDIGO) and the Risk, Injury, Failure, Loss, and End-stage (RIFLE) criteria of kidney disease [6, 7]. Although these diagnostic criteria are considered good predictors of nephropathy, they are neither sensitive nor specific mainly in the setting of early detection of AKI as they chiefly reflect functional changes in glomerular filtration capacity ignoring tubular function assessment.

Tubulointerstitial injury was found to precede glomerular injury during AKI with evidence of brush border detachment, effacement, and localized desquamation of tubular epithelial cells with inflammatory cell infiltration, and formation of casts containing an abundance of Tamm-Horsfall protein [8]. This inflammatory response further activates various inflammatory mediators including cytokines, chemokines, and prostanoid metabolites that act on renal vasculature and glomerular mesangial cells leading to hyperfiltration, matrix expansion, apoptosis, vasodilation, and further increase of mediators of cellular damage [9, 10].

Simple and sensitive biomarkers are being sought to help in prevention and early detection of renal tubular injury. Netrin- 1, a laminin-like protein, is a potent anti-inflammatory protein that plays an important protective role against metabolic dysfunction, insulin resistance, diabetes, and cardiovascular diseases [11–13]. Moreover, the kidney has one of the highest levels of netrin- 1 expression with studies showing increased urinary netrin- 1 secretion by proximal tubule epithelial cells in reaction to hypoxic or toxic injury, suggesting it is an early diagnostic marker of renal tubular injury [14]. In addition, administration of recombinant netrin- 1 before ischemia–reperfusion was found to reduce kidney injury and inflammation [15, 16]. Hence, this study assessed urinary netrin- 1 as an easy and sensitive biomarker of renal proximal tubular ischemia, inflammation, and injury.

Given the sparce data about renal tubular function during DKA and assuming its possible impairment potentially due to renal hypoperfusion and inflammation, this study aimed to assess renal tubular function in children and adolescents during DKA, its short-term outcomes and its relation to urinary netrin- 1 and renal perfusion indices, proposing these markers as potential early indicators and therapeutic targets for renal tubular injury in DKA.

## Materials and methods

### Study design

A prospective longitudinal study was conducted over 6 months at the Pediatrics and Adolescent Diabetes Unit (PADU), and Pediatric Dialysis and Nephrology Unit (PDNU), Faculty of Medicine, Ain Shams University. Participants were selected by simple random sampling.

### Sample size calculation

Assuming effect size difference in urinary netrin- 1 level in children with T1DM before and during DKA according to results from Uçaktürk and colleagues, using the G power program for sample size calculation, setting power at 80% and alpha error at 5%, a total sample size of 40 patients was found to be sufficient to achieve the study objective after considering a dropout rate of 10% [1].

### Study population

**Fig. 1:**
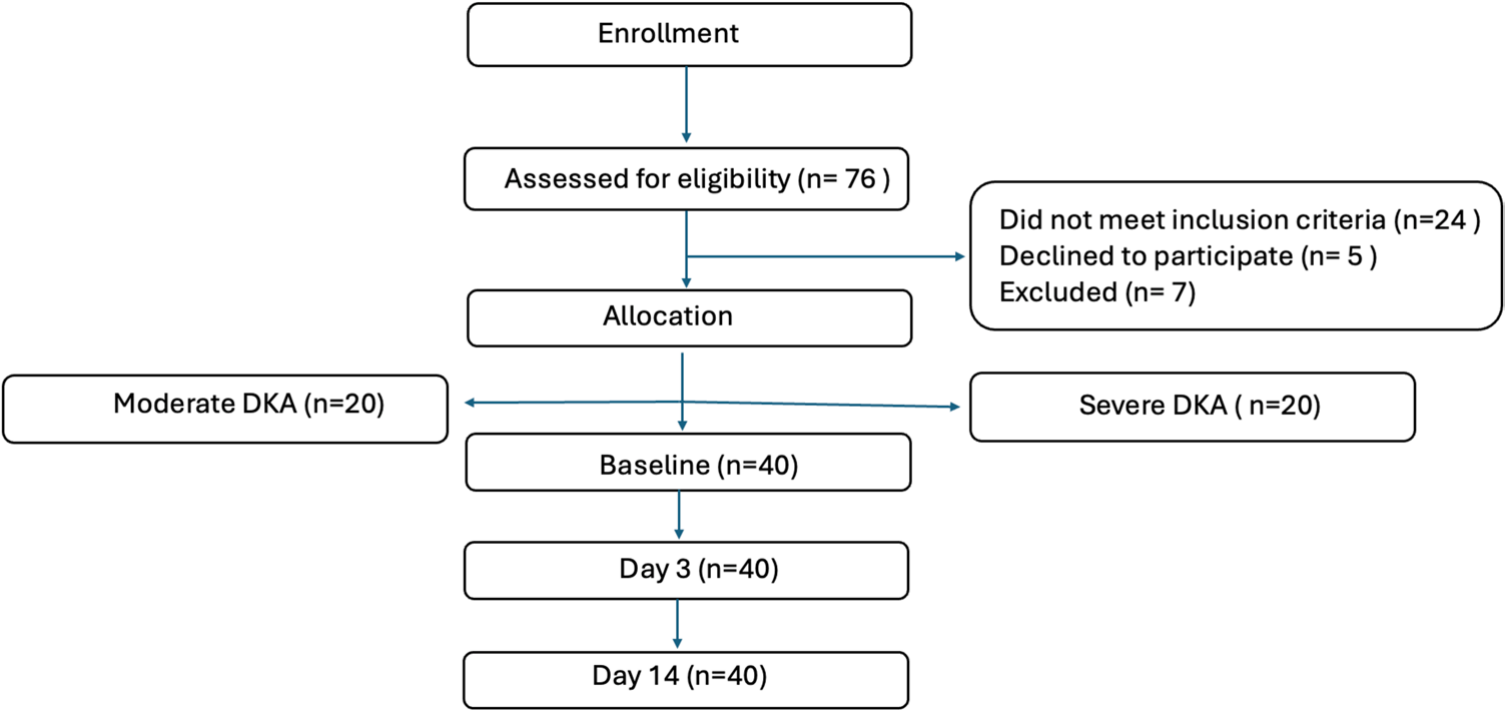
CONSORT flow chart according to the Standard Reporting of Observational Studies (STROBE) guidelines for the studied children and adolescents with moderate and severe DKA

Seventy-six children and adolescents with DKA were screened for eligibility. Diagnosis of DKA was made according to the criteria of the International Society of Pediatric and Adolescent Diabetes (ISPAD) 2022 [17]. Twenty-four patients did not meet inclusion criteria, 5 patients declined to participate, 7 patients were excluded, and 40 patients (20 moderate and 20 severe DKA) were enrolled (Fig. 1). Exclusion criteria included mild DKA, comorbid disorders affecting renal function (e.g., glomerulonephritis, hypertension), chronic kidney disease due to any cause including diabetic kidney disease, and positive COVID- 19 swabs.

### Ethical considerations

Approval was taken from the Institutional Review Board (IRB) and the Research Ethics Committee of the Faculty of Medicine, Ain Shams University (FMASU REC), with an approval number MS 314/2022 before the study commencement. Informed written consent for participation in the study and its publication was obtained from the parents or the legal guardians of the studied patients before participation. The study adhered to the Consolidated Standards of Reporting Trials Statement 2010 by the declaration of Helsinki [18].

### Procedures

All enrolled children and adolescents with DKA were subjected to detailed medical history with special emphasis on age, gender, diabetes duration, and insulin therapy (total insulin daily dose and type of insulin therapy). DKA severity was graded according to the criteria of the ISPAD 2022 [17].

### Each participant was assessed on baseline (during DKA), then reassessed on day 3 and day 14 from baseline including the following:

#### Clinical assessment

Auxological assessment was done, including weight in kilograms (Kg), height in centimeters (cm), and body mass index (BMI) in kg/m[2] with calculation of the z-scores according to age and gender [19]. Urine Output (UOP) was assessed as UOP in mL/kg/day. It was calculated using the equation (urine output in mL/kg/day). Polyuria was defined as UOP of more than 40–50 mL/kg/day over 24 h [20].

Systolic and diastolic blood pressures were measured using a mercury sphygmomanometer manually, two consecutive times, in the right arm, while the patient was relaxed and seated. The results were plotted on the relevant reference percentiles [21].

#### Biochemical assessment

##### Blood samples

About 5 ml of blood volume was withdrawn from each participant each time for assessment of:

- Random plasma glucose level, serum Na and K by SYNCHRON CX- 9 autoanalyzer (Beckman Coulter, USA).
- HbA1c via the Tina-QuantR HbA1c kit supplied by Roche Diagnostics on the Roche/Hitachi CobasR* c501 System (Roche Diagnostics International Ltd. CH- 6343 Rotkreuz, Switzerland) using turbidimetric inhibition immunoassay (TINIA).
- Complete blood count evaluated using an automated complete blood cell counter (Sysmex XT 1800i, Kobe, Japan).
- Kidney function tests: serum creatinine using the automated analyzer-based Jaffe method [22], urea using the Beckman BUN Analyzer with the calculation of the estimated glomerular filtration rate (eGFR) using the revised bedside Schwartz formula [23].
- Serum osmolality using the Smithlline-Gardner Eq. (2 X serum sodium) + [glucose, in mmol/L] + [urea, in mmol/L] which was found to have the best diagnostic accuracy in the pediatric population [24, 25].

##### Urine samples

Random urine samples were collected after 8 h of bed rest in sterile containers and then centrifuged at 2500 RPM for 20 min. The sample was divided into two parts, one was used immediately for urinary proteins, albumin, and creatinine assessment by an immuno-turbidimetric method using the Beckman Coulter AU 480 system (Beckman colter, Inc. 250 s. Kraemer Blvd. Brea, CA92821, USA). Urinary protein electrophoresis was performed on agarose gel by a Hellabio PE 10 kit (Hellabio, Thermi, Greece) on Helena scanner model 0280. Serum creatinine was measured simultaneously with the urine samples.

The other part of the urine sample was stored at − 20 °C and then processed at the end of the study by Enzyme-Linked Immuno-Sorbent Assay (ELISA) for urinary netrin- 1.

Proximal renal tubulopathy was indicated by the presence of tubular proteinuria defined as a urinary protein/creatinine ratio > 0.5 mg/mg for children < 2 years old and > 0.2 mg/mg for patients > 2 years old in the presence of a normal albumin-to-creatinine ratio suggesting the tubular origin of proteinuria [26]. This was further confirmed by urinary protein electrophoresis with predominant globulin excretion to confirm the tubular origin of proteinuria [27]. Albuminuria was defined as a urinary albumin/creatinine ratio (uACR) > 30 mg/gm creatinine [28]. Diagnosis of AKI was made according to the KDIGO criteria 2012 [6].

#### Radiological assessment

The renal duplex was performed by a single specialized radiologist using a GE Healthcare LOGIQ P9 ultrasound device equipped with a 6 to 12 MHz linear transducer to allow flow detection in pediatric patients. Before each assessment, the patients were nothing per os (NPO) for at least 4 h. Long and short axis 2D B-mode grayscale imaging was done first for both kidneys to determine the size, location, and echotexture with evaluation for any focal renal abnormality and corticomedullary differentiation. Color Doppler was then used to evaluate the blood flow in the proximal, mid, and distal renal arteries bilaterally and at the origin of each renal artery from the aorta. Using Spectral Doppler, the peak systolic velocity (PSV) was measured in the abdominal aorta at the level of the renal arteries, as well as in the renal artery origin, middle portion, and hilum [29]. The renal artery resistivity index (RI) was determined by dividing the difference between the PSV and end-diastolic velocity by the PSV (normal range is 0.5 to 0.7) and the renal artery pulsatility index (RAPI) was determined by dividing the difference between the PSV and end-diastolic velocity by the average velocity (normal range is 1 to 1.2) [30]***.***

### Data management and analysis

Data were collected, revised, coded, and tabulated using Statistical Package for Social Science (SPSS 27). For descriptive statistics mean, standard deviation (± SD), and range were used for parametric numerical data, median and interquartile range (IQR) were used for non-parametric numerical data, while frequency and percentage were used for non-numerical data. For analytical statistics, the Student *t*-test was used to assess the statistical significance of the difference between two study group means; Mann–Whitney test (*U* test) was used to assess the statistical significance of the difference of a non-parametric variable between two study groups; repeated measure ANOVA test was used to assess the statistical significance of the difference between more than two means measured at different time points; and chi-square test was used to examine the relationship between two qualitative variables. For comparing results during DKA, at D3 and D14, paired *t*-test was used to assess the statistical significance of the difference between two means measured twice for the same study group; Wilcoxon signed-rank test was used to assess the statistical significance of the difference of an ordinal variable (score) measured twice for the same study groups; and Friedman test was used to assess the statistical significance of the difference of a variable with multiple categories measured more than two times for the same study group.

Correlation analysis (using Spearman’s rho method) was used to assess the strength of association between two quantitative variables. The correlation coefficient denoted symbolically “r” defines the strength (magnitude) and direction (positive or negative) of the linear relationship between two variables. Univariate and multivariate logistic regression analysis was used to assess the most important factors associated with tubular proteinuria during DKA. The confidence interval was set to 95% and the margin of error accepted was set to 5%, so a *p*-value of < 0.05 was considered significant.

## Results

Forty children and adolescents (20 moderate and 20 severe) DKA were recruited from the emergency unit, PADU, Faculty of Medicine, Ain Shams University. Their mean age was 10.59 ± 2.17 years. Twenty percent of them were newly diagnosed with T1DM; while 80% had an established diagnosis of T1DM, with a median (IQR) duration of 3 (1.5–4) years.

### Renal tubulopathy and perfusion indices during DKA

On presentation, 16 (40%) of the studied children and adolescents with DKA had tubular proteinuria as confirmed by urinary protein electrophoresis, 11 (27.5%) had AKI according to the KDIGO classification of which 6 (15%) had both tubular proteinuria, and AKI and 5 (12.5%) had AKI alone (*p* = 0.427). The mean urine output of the studied children and adolescents with DKA at presentation was 14.06 ± 2.35 cc/kg/h, their mean serum K was 2.80 ± 0.27 mg/dl, their mean urinary protein/creatinine ratio was 0.8 ± 0.27 mg/mg, and their median (IQR) urinary albumin/creatinine ratio was 3.2 (0.65–3.44) mg/gm. As for renal perfusion, their mean renal pulsatility index was 0.87 ± 0.06, and their mean renal resistivity index was 0.87 ± 0.06, Table 1.

**Table 1:** Baseline characteristics of the studied children and adolescents during moderate and severe DKA

		Children and adolescents with DKA (*n* = 40)
Age (years)	Mean ± SD	10.59 ± 2.17
	Range	6–15
Gender	Female	36 (90.0%)
	Male	4 (10.0%)
Diabetes duration (years)	Median (IQR)	3 (1.5–4)
	Range	0–7
Weight (SDS)	Median (IQR)	− 0.33 (− 0.86–0.17)
	Range	− 1.72–0.92
Height (SDS)	Mean ± SD	2.12 ± 0.27
	Range	1.2–2.5
BMI (SDS)	Median (IQR)	1.15 (0.46–2.02)
	Range	− 1.51–2.4
Systolic blood pressure (mmHg)	Mean ± SD	100.61 ± 7.18
	Range	80–110
Systolic blood pressure (percentile)	Mean ± SD	62.06 ± 5.7
	Range	49–77
Diastolic blood pressure (mmHg)	Mean ± SD	63.03 ± 5.13
	Range	50–70
Diastolic blood pressure (percentile)	Mean ± SD	55.91 ± 16.4
	Range	5–83
Severity of DKA	Moderate	20 (50.0%)
	Severe	20 (50.0%)
UOP (cc/kg/h)	Mean ± SD	14.06 ± 2.35
	Range	10–18
Hematocrit (%)	Mean ± SD	39.6 ± 2.81
	Range	35–47
Serum osmolarity (mOsm/kg)	Mean ± SD	304.91 ± 8.05
	Range	287.7–318
eGFR (ml/min)	Mean ± SD	84.53 ± 31.45
	Range	77.4–210
Serum creatinine (mg/dl)	Mean ± SD	0.83 ± 0.22
	Range	0.4–1.2
BUN (mg/dl)	Mean ± SD	22.34 ± 6.46
	Range	14–41
Serum PH	Mean ± SD	7.11 ± 0.11
	Range	6.85–7.25
Serum bicarbonate	Mean ± SD	10.26 ± 2.97
	Range	4.6–15.4
Blood glucose (mg/dl)	Mean ± SD	508.22 ± 60.23
	Range	375–620
Serum Na (mmol/l)	Mean ± SD	132.88 ± 3.54
	Range	126–138
Serum K (mmol/dl)	Mean ± SD	2.80 ± 0.27
	Range	2.4–3.3
Urinary protein/creatinine ratio (mg/mg)	Mean ± SD	0.8 ± 0.27
	Range	0.4–1.4
Urinary albumin/creatinine ratio (mg/gm)	Median (IQR)	3.2 (0.65–3.44)
	Range	0.4–11.8
Urinary netrin- 1 (ng/ml)	Median (IQR)	723.08 (503.35–2098.48)
	Range	210.5–4223
Renal pulsatility index	Mean ± SD	0.87 ± 0.06
	Range	0.73–0.98
Renal resistivity index	Mean ± SD	0.61 ± 0.05
	Range	0.49–0.7

*BMI*, body mass index; *DKA*, diabetic ketoacidosis; *UOP*, urine output; *eGFR*, estimated glomerular filtration rate; *BUN*, blood urea nitrogen

Interestingly, no significant difference was found between those newly diagnosed with T1DM and those with an established diagnosis of T1DM regarding DKA severity (*p* = 0.376), proteinuria (*p* = 1.000), urinary netrin- 1 (*p* = 0.494), renal pulsatility (*p* = 0.648), and resistivity (*p* = 0.760) indices on presentation.

### Tubulopathy and DKA severity

Worth mentioning, children and adolescents with severe DKA were found to have significantly higher UOP (*p* = 0.002) and urinary protein/creatinine ratio (*p* = 0.001) than those with moderate DKA at presentation, Table 2. In addition, tubular proteinuria was found to be negatively correlated with serum PH (*p* = 0.016), and positively correlated with urine output and serum osmolarity (*p* = 0.014 and *p* = 0.012, respectively) with an independent association between DKA severity and tubular proteinuria on multivariate regression analysis, Tables 3 and 4.

**Table 2:** Relation of DKA severity with various clinical, laboratory, and radiological data on presentation

		Children and adolescents with DKA		Test of significance
		Moderate DKA (*n* = 20)	Severe DKA (n = 20)	*p*-value
Age (years)	Mean ± SD	10.65 ± 1.91	10.52 ± 2.44	0.858•
Gender	Female	18 (90.0%)	18 (90.0%)	1.000*
	Male	2 (10.0%)	2 (10.0%)	
Weight (SDS)	Median (IQR)	− 0.23 (− 0.95–0.17)	− 0.45 (− 0.81–0.19)	0.766 ≠
Height (SDS)	Mean ± SD	2.07 ± 0.34	2.16 ± 0.16	0.315•
BMI (SDS)	Median (IQR)	0.91 (0.31–2.06)	1.41 (0.62–1.91)	0.394 ≠
Systolic blood pressure (mmHg)	Mean ± SD	102.13 ± 7.66	99.1 ± 6.5	0.186•
Systolic blood pressure(percentile)	Mean ± SD	61.55 ± 6.16	62.56 ± 5.31	0.581•
Diastolic blood pressure (mmHg)	Mean ± SD	63.22 ± 5.63	62.83 ± 4.72	0.809•
Diastolic blood pressure(percentile)	Mean ± SD	53.25 ± 17.63	58.56 ± 15.04	0.312•
UOP (cc/kg/h)	Mean ± SD	12.98 ± 2.46	15.14 ± 1.69	**0.002**•
Hematocrit (%)	Mean ± SD	39.33 ± 3.4	39.95 ± 3.3	0.562•
eGFR (ml/min)	Mean ± SD	85.9 ± 23.53	83.16 ± 38.37	0.786•
Serum creatinine (mg/dl)	Mean ± SD	0.82 ± 0.21	0.84 ± 0.23	0.735•
Bun (mg/dl)	Mean ± SD	22.47 ± 7.63	22.2 ± 5.22	0.895•
Blood glucose (mg/dl)	Mean ± SD	457.9 ± 49.75	556.8 ± 61.17	** < 0.001**•
Serum Na (mmol/l)	Mean ± SD	132.6 ± 3.95	133.2 ± 4.1	0.640•
K (mmol/dl)	Mean ± SD	2.86 ± 0.27	2.74 ± 0.27	0.333•
Serum osmolarity (mOsm/kg)	Mean ± SD	305.29 ± 6.36	304.54 ± 9.6	0.774•
Urinary protein/creatinine ratio (mg/mg)	Mean ± SD	0.66 ± 0.22	0.93 ± 0.25	**0.001**•
Urinary netrin- 1 (ng/ml)	Mean ± SD	566.85 (407.08–1294.9)	836.9 (683.3–2322.5)	**0.047**•
Renal pulsatility index	Mean ± SD	0.84 ± 0.06	0.91 ± 0.05	** < 0.001**•
Renal resistivity index	Mean ± SD	0.58 ± 0.05	0.64 ± 0.04	** < 0.001**•

*BMI*, body mass index; *DKA*, diabetic ketoacidosis; *UOP*, urine output; *eGFR*, estimated glomerular filtration rate; *BUN*, blood urea nitrogen

Mean and SDS were used in case of normally distributed variables and median and IQR in case of non-normally distributed variables

•Student *t*-test of significance (t); ≠ Mann–Whitney test of significance; *chi-square test of significance

*p* < 0.05: significant (bold)

**Table 3:** Correlation of tubular proteinuria during DKA and various clinico-laboratory and radiological parameters

	Protein/creatinine ratio (mg/mg)	
	r	*p*-value
Serum PH	− **0.379**	**0.016**
Age (years)	0.189	0.242
Diabetes duration (years)	0.267	0.096
Weight (SDS)	0.086	0.597
Height (SDS)	0.202	0.212
BMI(SDS)	− 0.184	0.257
Systolic blood pressure (mmHg)	− 0.093	0.566
Systolic blood pressure (centiles)	− 0.248	0.122
Diastolic blood pressure (mmHg)	0.087	0.591
Diastolic blood pressure (centiles)	− 0.022	0.891
Urine output (cc/kg/h)	**0.439**	**0.014**
Blood glucose (mg/dl)	**0.302**	**0.049**
Hematocrit (%)	0.198	0.221
Serum osmolarity (mOsm/kg)	**0.489**	**0.012**
eGFR (ml/min)	− 0.067	0.683
Serum creatinine (mg/dl)	− 0.001	0.995
BUN (mg/dl)	0.079	0.628
HbA1c (%)	0.198	0.221
Serum Na (mmol/l)	− 0.252	0.117
Renal pulsatility index (average)	**0.612**	** < 0.001**
Renal resistive index (average)	**0.368**	**0.020**
Urinary netrin- 1 (ng/ml)	**0.430**	**0.006**

Spearman’s rho method, *p* < 0.05: significant (bold)

*DKA*, diabetic ketoacidosis; *eGFR*, estimated glomerular filtration rate; *BMI*, body mass index; *BUN*, blood urea nitrogen; *HbA1c*, glycated hemoglobin

**Table 4:** Univariate and multivariate linear regression analysis for the most important predictors of tubulopathy during DKA among children and adolescents with T1DM

	Unstandardized coefficients		Standardized coefficients	*t*	Significance
		± *SE*	Beta		
(Constant)	6.308	9.243		0.682	0.506
Severity of DKA	− 0.325	0.271	− 0.329	− 3.197	**0.005**
Serum PH	− 1.085	1.150	− 0.294	− 0.944	0.361
Urinary netrin- 1 (ng/ml)	0.805	0.309	0.175	0.657	0.522
Renal pulsatility index	− 0.310	2.797	− 0.067	− 2.111	**0.013**
Renal resistivity index	4.090	3.046	0.694	2.343	**0.021**

*DKA*, diabetic ketoacidosis; *T1DM*, type 1 diabetes mellitus

*β*, regression coefficient; *SE*, standard error

*p* < 0.05: significant (bold)

### Renal perfusion, tubulopathy, and DKA severity

Interestingly, children and adolescents with severe DKA were found to have significantly higher renal pulsatility and resistivity indices than those with moderate DKA (*p* < 0.001), Table 2. In addition, renal pulsatility and resistivity indices were positively correlated with tubular proteinuria at presentation (*p* < 0.001 and *p* = 0.02, respectively), Table 3. Moreover, tubular proteinuria was independently associated with renal pulsatility (*p* = 0.013) and resistivity (*p* = 0.012) indices on multivariate regression analysis, Table 4.

### Urinary netrin- 1, tubulopathy, and DKA severity

**Fig. 2:**
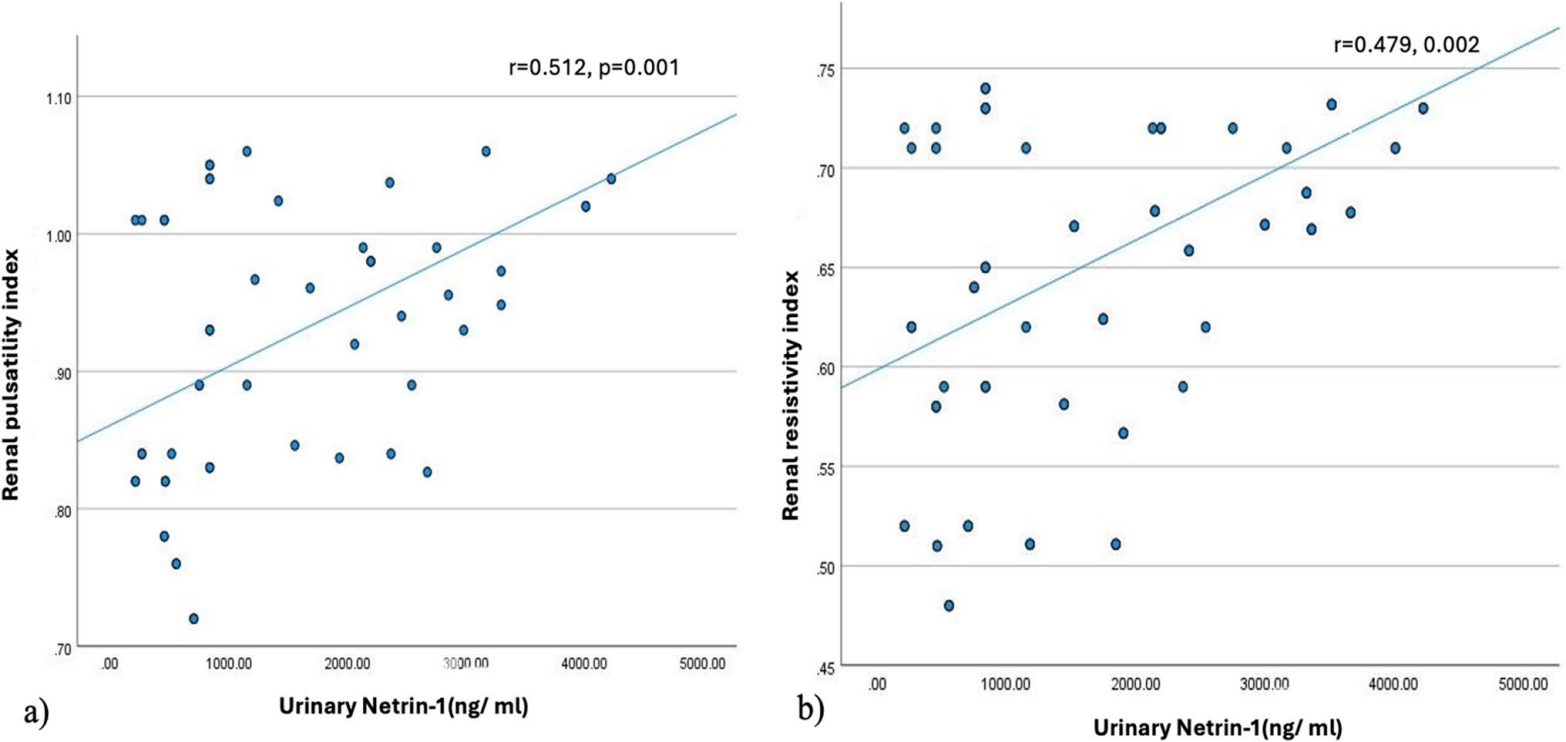
**a** Correlation between renal pulsatility index and urinary netrin- 1 Netrin- 1 in children and adolescents during DKA. **b** Correlation between renal resistivity index and urinary netrin- 1 Netrin- 1 in children and adolescents during DKA

Urinary netrin- 1 was found to be positively associated with DKA severity (*p* = 0.047), and correlated with tubular proteinuria (*p* = 0.006), Table 3. Moreover, urinary netrin- 1 was found to be positively correlated with renal pulsatility and resistivity indices (*p* = 0.001 and *p* = 0.002, respectively), Fig. 2 and to be independently associated with renal pulsatility and resistivity indices on multivariate regression analyses (*p* = 0.006 and *p* = 0.037, respectively).

### Tubulopathy fate after DKA

**Fig. 3:**
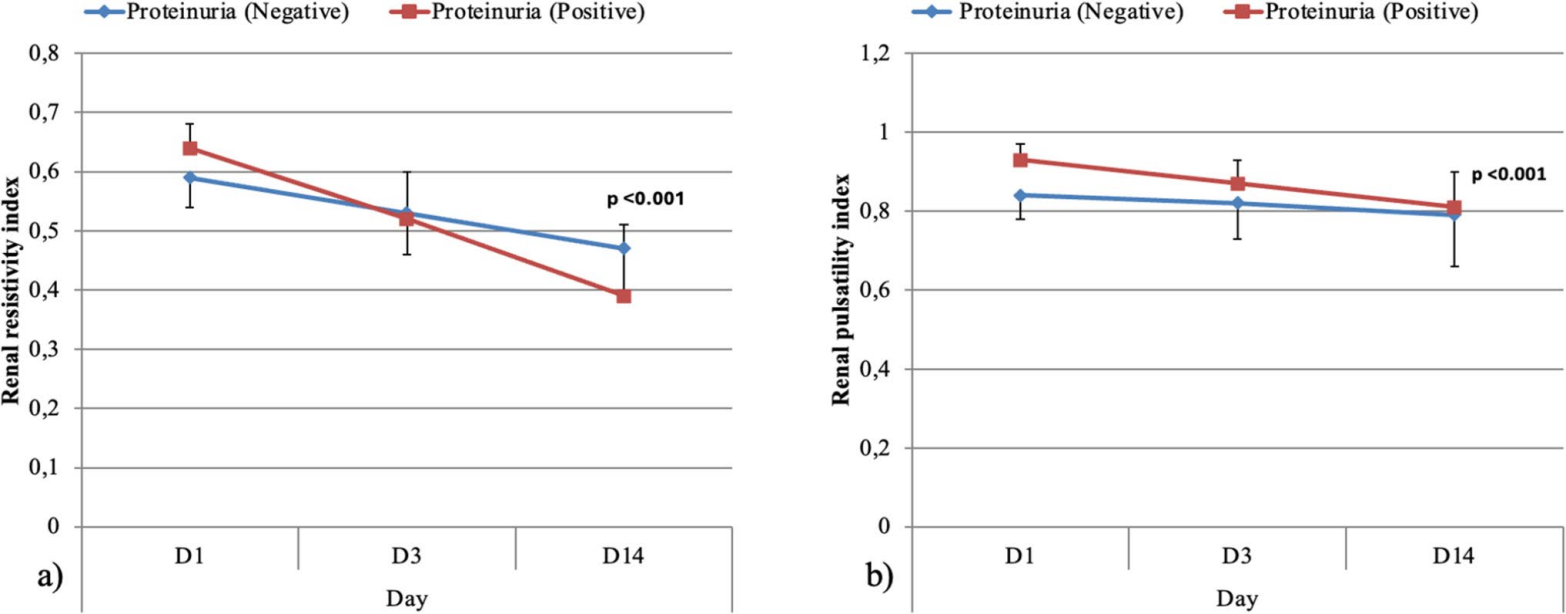
**a** Changes in mean renal resistivity index at presentation, day 3 and day 14 among the studied children and adolescents with DKA with and without tubular proteinuria. **b** Changes in mean renal pulsatility index at presentation, day 3 and day 14 among the studied children and adolescents with DKA with and without tubular proteinuria

**Fig. 4:**
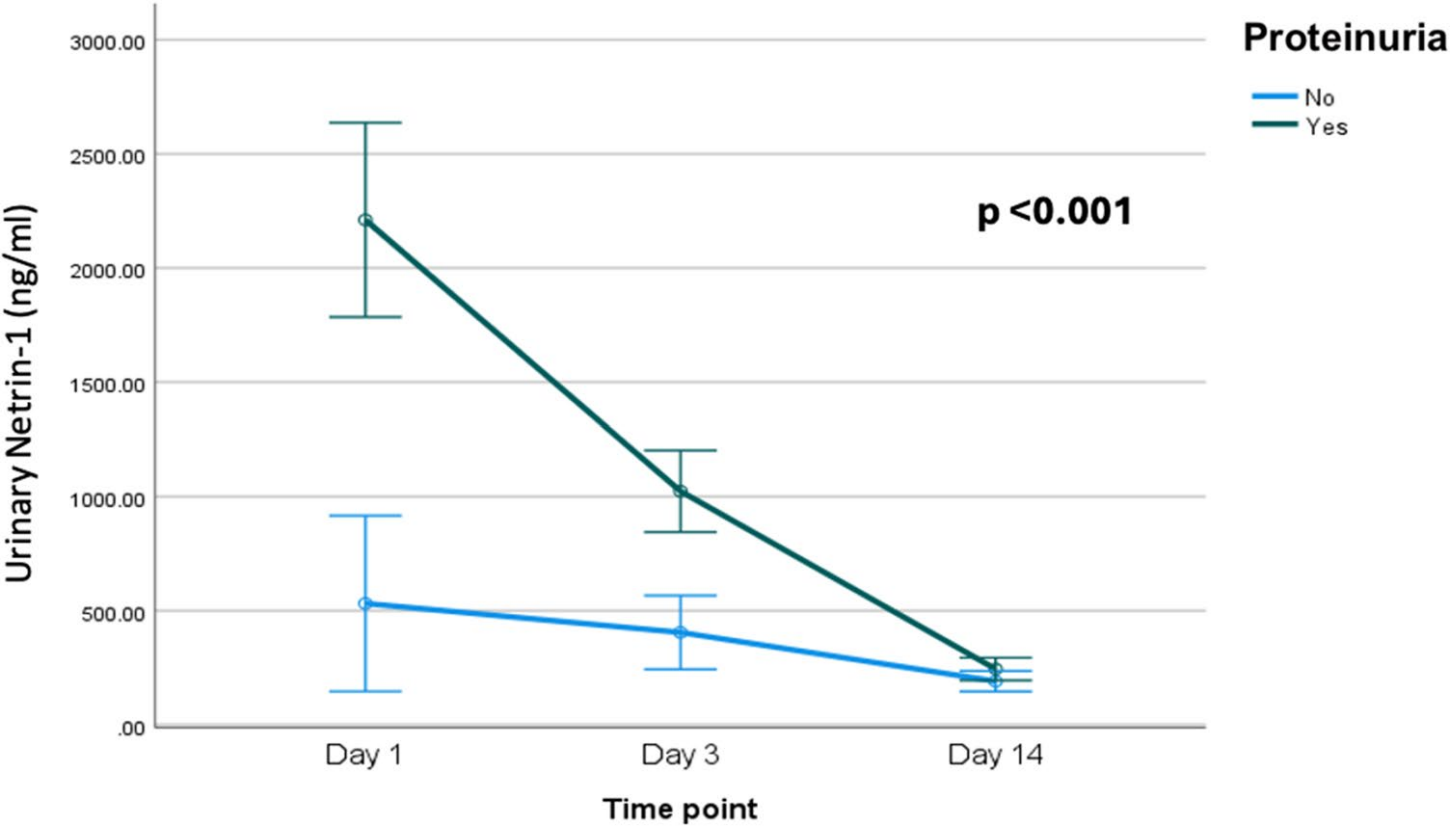
Changes in urinary netrin- 1 Netrin- 1 level at presentation, day 3 and day 14 among the studied children and adolescents with DKA with and without tubular proteinuria

Upon serial follow-up of the studied children and adolescents with DKA, significant improvement was found in the urine output, serum potassium, renal pulsatility, and resistivity indices on day 3 with complete normalization of the urinary protein/creatinine ratio, urine output, serum osmolarity, and serum K on day 14 (< 0.001), Table 5 and Figs. 3, 4.

**Table 5:** Serial follow of the clinical, laboratory, and radiological data of the studied children and adolescents with moderate and severe DKA at presentation, days 3 and day 14

		Day 1	Day 3	Day 14	Test value	*p*-value	Percent change
UOP (cc/kg/h)	Mean ± SD	14.06 ± 2.35	3.31 ± 0.99	2.09 ± 0.17	746.455•	** < 0.001**	− 86% (− 86.7 to − 81.3%)
	Range	10–18	2–6	2–2.6			
Hematocrit (%)	Mean ± SD	39.6 ± 2.81	37 ± 2.42	37.16 ± 1.68	21.175•	** < 0.001**	− 5.3% (− 11.2 to − 1.4%)
	Range	35–47	32.8–42	34–42			
eGFR (ml/min)	Mean ± SD	84.53 ± 31.45	123.17 ± 23.29	137.46 ± 36.58	44.178•	** < 0.001**	33% (0–92.4%)
	Range	50.4–210	84.1–177.1	100.8–280.5			
Serum creatinine (mg/dl)	Mean ± SD	0.83 ± 0.22	0.56 ± 0.17	0.54 ± 0.07	52.534•	** < 0.001**	− 25% (− 42.7–0%)
	Range	0.4–1.4	0.4–1	0.3–0.7			
Bun (mg/dl)	Mean ± SD	22.34 ± 6.46	13.66 ± 4.2	11.84 ± 1.4	87.830•	** < 0.001**	− 42.7% (− 53.9 to − 28.2%)
	Range	14–41	8–28	8–15			
Blood glucose (mg/dl)	Mean ± SD	508.22 ± 60.23	247.49 ± 10.08	125.04 ± 18.38	1085.753•	** < 0.001**	− 76.9% (− 79.8 to − 70.4%)
	Range	375–620	234–275	97–157			
Serum Na (mmol/l)	Mean ± SD	132.88 ± 3.54	134.1 ± 3.01	134.58 ± 2.39	4.667•	**0.017**	1.8% (− 0.7–3.9%)
	Range	126–138	123–139	128–139			
Serum K (mmol/dl)	Mean ± SD	2.80 ± 0.27	3.59 ± 0.32	4.09 ± 0.52	9.535•	** < 0.001**	47.7%
	Range	2.4–3.3	3.1–4.4	3.4–5.5			(20.5–73.2%)
Serum osmolarity (mOsm/kg)	Mean ± SD	304.91 ± 8.05	290.78 ± 2.2	282.78 ± 1.2	213.944	** < 0.001**	− 7.7% (− 8.9 to − 6.4%)
	Range	287.7–318	287.8–296	280.9–285.01			
Urinary protein/creatinine ratio (mg/mg)	Mean ± SD	0.8 ± 0.27	0.3 ± 0.12	0.03 ± 0.01	276.71•1	** < 0.001**	− 93.1% (− 96.2 to − 83.6%)
	Range	0.4–1.4	0.2–0.8	0.01–0.06			
Urinary netrin- 1 (ng/ml)	Median (IQR)	723.08 (503.35–2098.48)	764.33 (518.73–1159)	180.3 (153.48–255.43)	54.350 ≠	** < 0.001**	− 73.6% (− 86.6 to − 46.9%)
	Range	210.5–4223	243.6–1700	115.3–500.5			
Renal pulsatility index	Mean ± SD	0.87 ± 0.06	0.82 ± 0.06	0.8 ± 0.11	14.315•	** < 0.001**	− 11.35% (− 17.55 to − 2.9)
	Range	0.73–0.98	0.71–0.99	0.67–1.3			
Renal resistivity index	Mean ± SD	0.61 ± 0.05	0.56 ± 0.04	0.43 ± 0.11	46.226•	** < 0.001**	− 12.75% (− 13.75 to − 4.2%)
	Range	0.49–0.7	0.48–0.62	0.26–0.64			

*DKA*, diabetic ketoacidosis; *UOP*, urine output; *eGFR*, estimated glomerular filtration rate; *BUN*, blood urea nitrogen; *IQR*, interquartile range; *SD*, standard deviation

•Repeated measures ANOVA; ••paired *t*-test; *chi-square test; ≠ Friedman test

*p* < 0.05: significant (bold)

## Discussion

Glomerular injury has received significant attention in diabetic kidney disease, yet, tubular proteinuria is thought to precede glomerular microalbuminuria in the course of diabetic nephropathy, suggesting tubulopathy as an early manifestation of diabetic kidney disease [31]. Accumulating evidence suggests a crucial role for tubulopathy in ischemic AKI, with both proximal tubules and thick ascending limbs proposed to act as sensors, effectors, and injury recipients of ischemic AKI stimuli [4]. However, the frequency, pathophysiological determinants, and short-term outcomes of tubulopathy during DKA remain poorly understood.

In the current study, 40% of the studied children and adolescents had tubulopathy and 27.5% had AKI during DKA, which goes in line with Hursh and colleagues who found AKI in 64.2% of children with DKA, Piani and coworkers who demonstrated renal tubular injury during DKA, and Marzuillo and colleagues who reported tubulopathy in 100% of children with DKA [10, 32, 33]. The difference in the frequencies could be attributed to the difference in DKA severity among the studied cohorts. However, the frequency is high in all these studies which necessitates unraveling the mechanisms underlying tubulopathy and AKI during DKA aiming to prevent them.

Several mechanisms were proposed for the DKA-associated tubulopathy including insulinopenia, dehydration, ketosis, neurohormonal activation, and inflammation. One suggested mechanism is the reduced glucose uptake into renal tubular cells due to marked insulin deficiency during DKA, resulting in energy deficiency and renal tubular dysfunction [34]. Another mechanism is the free fatty acids increase during DKA, due to accelerated lipolysis, resulting in increased blood ketones which may directly damage renal tubular cells [35]. Moreover, acute hyperglycemia is thought to cause renal tubular damage through increased reactive oxygen species (ROS) in renal tubular epithelial cells aggravating mitochondrial damage and renal tubular cell apoptosis [36]. In addition, renal hypoperfusion and impaired renal microcirculation during DKA are thought to cause ischemic renal tubular injury [37]. Thus, identifying the exact pathomechanistic causes of renal tubular injury during DKA and trying to prevent them is of utmost importance.

In the present study, a significant positive correlation was found between tubular proteinuria and hyperglycemia during DKA. This goes in concordance with Wang et al. who reported dose-dependent renal tubular injury with acute hyperglycemia in rats [36]. This could be because proximal tubular epithelial cells cannot decrease glucose transport when exposed to hyperglycemia which renders them vulnerable to hyperglycemia-induced damage through increased ROS and mitophagy inhibition [35].

Notably, tubular proteinuria was found to be independently correlated with DKA severity among the studied children and adolescents. This goes in line with Piani and colleagues who reported higher acute kidney injury biomarkers among participants with moderate and severe DKA than those with mild DKA across all time points [32]. Similarly, Marzuillo and colleagues observed that patients with renal tubular injury at T1DM presentation were more severely affected in terms of dehydration and acidosis than patients without tubular injury [33]. This could be because as the DKA severity increases, the degree of hyperglycemia, oxidative stress, and inflammation increases leading to more renal tubular damage.

Impaired renal tubular microcirculation had been observed in healthy individuals with acute hyperglycemia [38]. This is attributed to the structural characteristics of the renal tubular microcirculation rendering renal tubular cells vulnerable to hypoperfusion [36]. This goes in concordance with the current study, where proteinuria was found to be independently associated and correlated with renal hypoperfusion indices.

Interestingly, glomerular functions were not significantly affected among the studied children and adolescents during DKA despite the significant tubular affection as shown by the urinary microalbumin, serum creatinine, and eGFR levels. This goes in line with Marzuillo and colleagues, who reported isolated acute tubular injury among 30% of patients at T1DM onset without AKI. They reported that none of the participants with tubular injury showed serum creatinine values reaching the KDIGO AKI criteria suggesting that tubular injury precedes glomerular injury [33] which agrees with known pathophysiological mechanisms indicating that acute tubular damage causes a fall in glomerular filtration rate to compensate for failure to reabsorption of filtered solute [39].

Netrin- 1 a laminin-related protein is known to be highly expressed in renal tubular epithelial cells. Under physiological conditions, netrin- 1 cannot be filtered by the glomeruli, having a molecular mass of 72 kDa, and is located in the peritubular capillaries [40]. Urinary netrin- 1 excretion was found to be markedly elevated by tubular epithelial cells in various acute and chronic kidney diseases including diabetic nephropathy and acute renal ischemia; however, its pathophysiological role in DKA-induced tubulopathy is still unknown [41]. In the current study, urinary netrin- 1 was positively associated with DKA severity, and correlated with tubulopathy and renal hypoperfusion indices. This goes in line with previous studies that reported a marked increase of urinary netrin- 1 by proximal tubular epithelial cells in diabetes-induced renal tubular injury [42, 43]. Similarly, Uçaktürk and coworkers demonstrated a significant increase in urinary netrin- 1 excretion in children with diabetes that is correlated with HbA1c indicating renal proximal tubular affection and suggesting that tubular injury markers might be affected by short-term fluctuations in blood glucose levels [1]. In addition, netrin- 1 was found to be overexpressed by the renal proximal tubular epithelial cells in ischemic renal injury and netrin- 1 overexpression in transgenic mice protects the kidneys from ischemia–reperfusion injury by increasing vascular proliferation, suppressing apoptosis, reducing cytokine expression, and reducing oxidative stress [16]. Hence, an important protective role for netrin- 1 in DKA-induced tubulopathy is postulated. Acute renal ischemia and hyperglycemia during DKA stimulate netrin- 1 excretion by the kidney which has a vascular protective, anti-inflammatory, and antiproteinuric effect on renal tubules trying to spare the tubules. Thus netrin- 1 could serve as a potential therapeutic target for DKA-induced tubulopathy.

In the current study, complete resolution of the tubulopathy and renal ischemia markers was demonstrated among the affected children and adolescents by 14 days post-DKA. This goes in agreement with Piani and colleagues who observed complete resolution of the renal tubular injury in a prospective cohort study by 3 months follow-up [32]. Similarly, Kumagai and coworkers found that proximal renal tubular dysfunction was alleviated shortly after insulin treatment in all patients with DKA. They suggest that insulin treatment improves glucose uptake into renal tubular cells, restores ATP production, and decreases ketone body production, resulting in the relief of renal tubular dysfunction [35]. However, further long-duration longitudinal studies are needed to explore the long-term effects of DKA on renal tubular functions and the potential role of netrin- 1 as a therapeutic target.

## Conclusion

Transient renal tubulopathy is seen during moderate and severe DKA manifested by proteinuria, polyuria, and hypokalemia that is completely reversible by day 14 post-DKA. The degree of this tubulopathy is associated with DKA severity, renal hypoperfusion, and increased urinary netrin- 1. Hence, maintaining renal perfusion during DKA is crucial to avoid renal tubular injury. Careful monitoring of serum electrolytes, proteins, and urine output is recommended for children and adolescents for 14 days post DKA with correction of any electrolytes or body fluid volume derangements. Netrin- 1 could serve as a potential therapeutic target for DKA-associated tubulopathy.

## Strength and limitations

Strengths points of this study include its prospective design, which allowed serial assessment of renal tubular functions together with renal perfusion indices and urinary netrin- 1. However, it has some limitations including the short duration of follow-up (14 days), the exclusion of those without DKA and with mild DKA, and the relatively small sample size. Hence, larger longer duration studies including children and adolescents without DKA and with mild DKA are needed to identify the renal tubular functions in those without DKA and with mild DKA and the long-term sequelae of DKA-associated tubulopathy.

## Data Availability

Data will be available upon request from the corresponding author.
